# Heart rate markers for prediction of fetal acidosis in an experimental study on fetal sheep

**DOI:** 10.1038/s41598-022-14727-4

**Published:** 2022-06-23

**Authors:** Louise Ghesquière, C. Ternynck, D. Sharma, Y. Hamoud, R. Vanspranghels, L. Storme, V. Houfflin-Debarge, J. De Jonckheere, C. Garabedian

**Affiliations:** 1 Univ. Lille, CHU Lille, ULR 2694-METRICS-Evaluation des technologies de santé et des pratiques médicales, 59000 Lille, France; 2 Department of Obstetrics, CHU Lille, 59000 Lille, France; 3 Department of Biostatistics, CHU Lille, 59000 Lille, France; 4 Department of Pediatric Surgery, CHU Lille, 59000 Lille, France; 5 Department of Neonatology, CHU Lille, 59000 Lille, France; 6 CHU Lille, CIC-IT 1403, 59000 Lille, France; 7 Department of Obstetrics, CHU Lille, Avenue Eugène Avinée, 59037 Lille Cedex, France

**Keywords:** Computer science, Information technology, Scientific data, Statistics

## Abstract

To overcome the difficulties in interpreting fetal heart rate (FHR), several tools based on the autonomic nervous system and heart rate variability (HRV) have been developed. The objective of this study was to use FHR and HRV parameters for the prediction of fetal hypoxia. It was an experimental study in the instrumented fetal sheep. Repeated umbilical cord occlusions were performed to achieve severe acidosis. Hemodynamic parameters, ECG, and blood gases were analyzed. The variables used were heart rate baseline, HRV analysis (RMSSD, SDNN, LF, HF, HFnu, Fetal Stress Index (FSI), …), and morphological analysis of decelerations. The gold standard used to classify hypoxia was the fetal arterial pH (pH < 7.10). Different multivariable statistical methods (logistic regression and decision trees) were applied for the detection of acidosis. 21 lambs were instrumented. A total of 130 pairs of FHR/fetal pH analysis were obtained of which 29 in the acidosis group and 101 in the non-acidosis group. After logistic regression model with bootstrap resampling and stepwise backward selection, only one variable was selected, FSI. The AUC of FSI alone in this model was 0.81 with a sensitivity of 0.66, specificity of 0.88, PPV of 0.61, and NPV of 0.90 considering a threshold of 68. Decision trees with CHAID and CART algorithms showed a sensitivity of 0.48 and 0.59, respectively, and a specificity of 0.94 for both. All employed methods identified HRV variables as the most predictive of acidosis. The primary variables selected automatically were those from the HRV. Supporting the use of FHRV measures for the screening of fetal acidosis during labour is interesting.

## Introduction

Fetal well-being assessment during labor is a major concern, with fetal hypoxia remaining a significant cause of neonatal morbidity and mortality^1^. Assessment is based currently on the fetal heart rate (FHR), as recorded via a cardiotocograph (CTG)^2,3^. However, even if used continuously throughout labor, it does not offer a satisfactory evaluation of fetal oxygenation or the neonatal risk. It is both imperfect and subjective, with significant operational variability in CTG interpretation^4^, despite the existence of classification templates to assist in the FHR analysis^5^.

To compensate for this variability in FHR interpretation, computerized and automated FHR analysis systems have been developed, not only to improve the detection of fetal hypoxia but also to reduce the rate of medical intervention^6,7^. These various systems are associated with artificial intelligence (AI) and/or machine learning (ML). They consider several FHR-related parameters such as variability and acceleration/deceleration rates. For example, the INFANT system (INFANT System, K2 Medical Systems, Plymouth, UK) provides an automated interpretation of FHR using neural-network algorithms for classification^8^. In 2017, a randomized study of 47,062 patients compared the use of the INFANT system against visual FHR analysis for neonatal-outcome prediction^9^, but found that there was no significant difference with respect to neonatal morbidity and mortality between the two groups. Ayres de Campos et al. developed the SisPorto automated FHR analysis system (Omniview-SisPorto 4.0, Speculum, Lisbon, Portugal)^10^. They showed that the system was effective in predicting a low Apgar score at birth but not so in the detection of fetal acidosis^6^. In a 2017 randomized clinical trial of 7730 patients, they compared the use of the SisPorto 4.0 system against visual FHR analysis^11^. They found that rates of metabolic acidosis were low for both the SisPorto group (0.40%) and the visual group (0.58%), but the difference was not statistically significant. In a retrospective before-and-after study comprising 38,466 deliveries, Lopes-Pereira et al. found a significant decrease in the number of cases per 1000 births of hypoxo-ischemic encephalopathy (HIE) after the introduction of the SisPorto system, together with a reduction in cesarean deliveries^12^.

The use of these tools has been encouraging, although they have not been shown to be effective in the detection of fetal hypoxia. They have focused on the analysis of FHR via CTG but have not yet consider heart rate variability (HRV). HRV analysis may be an interesting option for fetal-hypoxia screening, and the addition of HRV analysis markers in these automated models could improve the screening for cases at risk of acidosis during labor.

The main objective of this study is to investigate the feasibility of the prediction of fetal acidosis using FHR and HRV parameters. Though it may differ from human fetuses, this feasibility study was performed on an experimental standardized animal model for fetal acidosis. Such a model allows obtaining a continuous and progressive decrease of pH for a better analysis of the FHR and HRV parameters prediction abilities.

## Materials and methods

To obtain a database of FHR recordings that allow for HRV analysis, an experimental study involving 21 fetal sheep was set up within the University Hospital Department of Experimental Research at the Lille Faculty of Medicine from 2017 to 2019. From these 21 fetal sheep, a total of 170 measurements of FHR coupled with pH were obtained. Because of signal quality problems only 130 measurements were studied.

### Ethics

The anesthetic, surgical, and experimentation protocols were consistent with recommendations from the Ministry of Higher Education, Research, and Innovation, with the study being approved by the Animal Experimentation Ethics Committee of Nord—Pas de Calais, France (CEEA #2016121312148878). This manuscript is compliant with the ARRIVE guidelines for reported animal research^13^. All methods were performed in accordance with the relevant guidelines and regulations. The sheep used in this study didn’t the client-owned animals.

### Surgical preparation

Near-term pregnant sheep (breed "Ile de France," INRA, Val de Loire, France) with a gestational age of 124 ± 1 days (term = 145 days) underwent our previously described surgical procedure^14–16^. For anesthesia, the ewes had a vascular infusion of 500 ml of Ringer's lactate, followed by premedication with 0.3 ml intravenous sedaxylan (xylazine 20 mg/ml, Dechra, the Netherlands). Induction was via 5% isoflurane before intubation and anesthesia was maintained with 2% isoflurane. A midline abdominal incision was made to expose the uterus and the fetus was partially exteriorized for instrumentation. Two catheters (4 Fr diameter, Arrow, USA) were placed in the fetal axillary arteries, one on each side. Four electrocardiograph (ECG) electrodes (Mywire 10, Maquet, Rastatt, Germany) were placed on the fetal intercostal muscles to record the fetal ECG. An additional catheter was placed into the amniotic sac to measure pressure within the amniotic space. An inflatable silicone occluder (OC16, In Vivo Metric, Healdsburg, CA) was placed around the umbilical cord and the volume of saline solution required to achieve complete occlusion was determined. If the ewe had multiple fetuses, only one was instrumented and included in the experiment. (The fetus that was easiest to access during surgery was chosen to minimize twisting of the uterus).

### Experimental protocol

The experimental protocol began 72 h after the surgery. Upon arrival of the ewe in the experimental room, an acclimatization period of one hour was performed in the presence of the primary investigators before start the protocol. During this phase, called baseline phase, fetal arterial catheters and an intra-amniotic catheter were connected via pressure sensors (Pressure Monitoring Kit1, Baxter, France) to a multiparametric monitor (Monitor Merlin, Hewlett Packard, Palo Alto, CA, USA). The arterial blood pressure (ABP) was measured from the blood pressure phasic signal and referenced to the intra-amniotic pressure (IAP). ECG electrodes were connected to the multiparametric monitor. Hemodynamic data were recorded continuously throughout the experiment*.* When the ewe was acclimated, after a 1-h baseline period, the protocol started: repetitive umbilical cord occlusions (UCOs) were performed by injecting an isotonic solution into the occluder to obtain a total occlusion for 1 min (min). The protocol was divided into three 1-h phases, as described by Prout et al.^17^. UCOs were repeated every 5 min during the first phase (phase A), every 3 min during the second phase (phase B), and every 2 min during the third phase (phase C). At the end of the baseline phase and every 20 min during the procedure, a 5-min period without UCOs, called stable period was observed to enabled the evaluation of the HRV markers and gasometric parameters. The protocol was stopped if the pH dropped below 7.00 to avoid the risk of fetal death during the protocol. Euthanasia was administered at the end of the experimental procedure, 2 days after, or earlier in case of in utero fetal death or death during surgery. Euthanasia was carried out by intravenous injection of 6 ml/50 kg of T61 (1 ml contains embutramide 200 mg + mebezonium 26.92 mg + tetracaine 4.39 mg, MSD, France).

### Measurement of gasometric and hemodynamic parameters

Fetal blood gas measurements were performed on arterial blood samples taken from one of the arterial catheters during the baseline phase and after the end of the last UCO in each of the phases A, B, and C. Gasometric parameters (pH, PaO_2_, PCO_2_, base excess (BE), plasma lactate) were then measured with the iSTAT1 blood analyzer (iSTAT1 System, Abbott Point of Care Inc, Princeton, NJ, USA) using CG4 + cartridges.

All the hemodynamic data were recorded on computer using the Physiotrace^®^ software package^18^. The FHR, mean ABP (MAP), and mean IAP were read from the multiparametric scope at the same time. The MAP was corrected by subtracting the IAP (corrected MAP = observed MAP − observed mean IAP). We also noted the corrected MAP and FHR nadir during the last occlusion of each phase. Our experimental design involved between one and nine measurements (one every 20 min) of gasometric/HRV data per fetus. Acidosis was defined as an arterial pH < 7.10, and two pH groups, "acidosis" and "no acidosis", were created according to the presence or absence of acidosis, respectively. So, for any animal, the earlier datapoints (when animals were non-acidotic, pH ≥ 7.10) were included in the “non-acidotic” group, and the later timepoints (pH < 7.10) were in the “acidotic” group.

### Acquisition of the FHR and processing of the ECG signal

Three of the four ECG electrodes were connected to a monitor (Merlin monitor, Hewlett-Packard, Palo Alto, CA, USA), enabling the collection of the ECG signal and the calculation of the mean (FHRmean), minimum (FHRmin), and maximum (FHRmax) FHR signals. ECG signals were recorded by a Physiotrace data acquisition card (Physiotrace, Estaris Monitoring, Lille, France).

#### Analysis of HRV parameters

**Figure 1:**
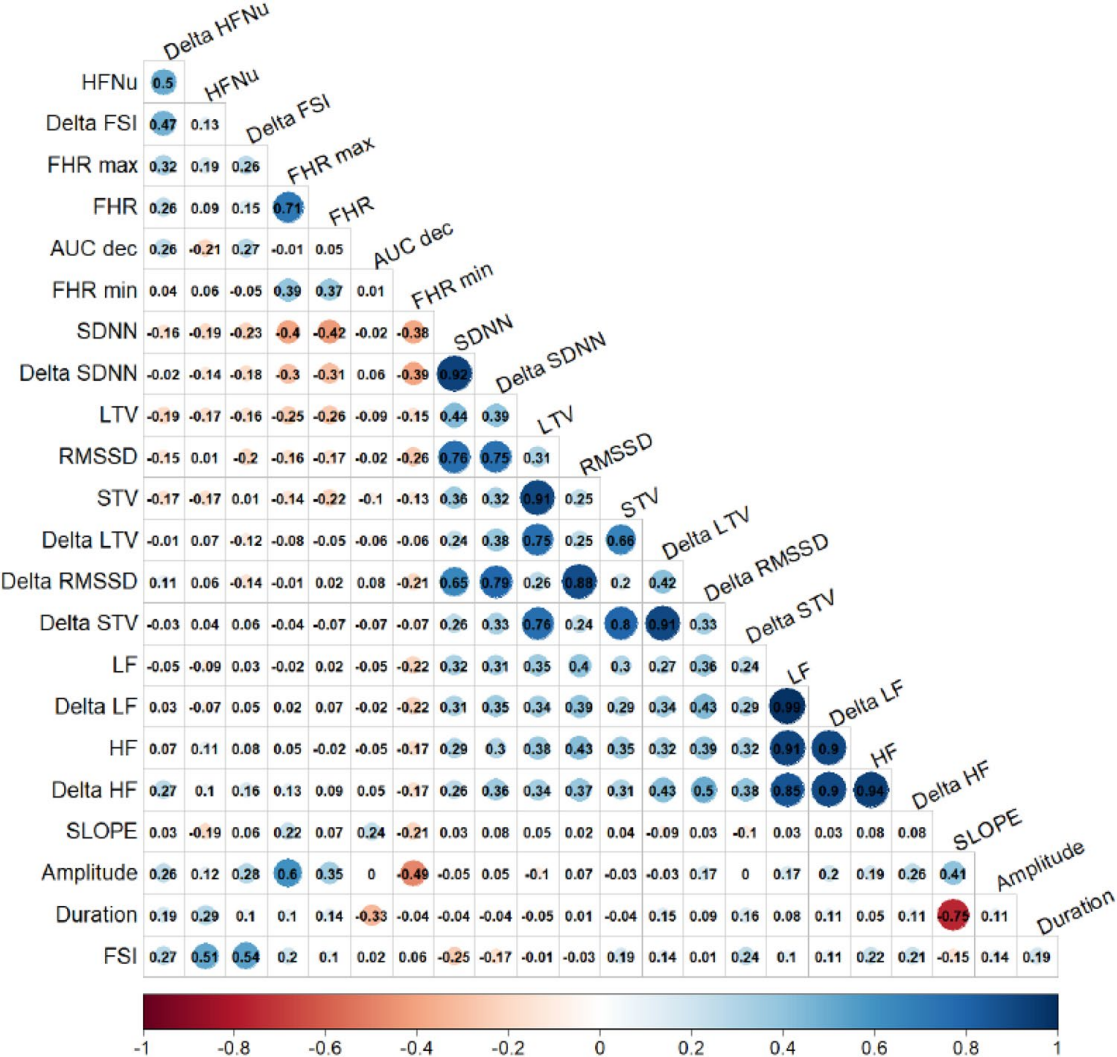
Pearson correlation coefficients of the 23 variables. Variables were highly correlated if the coefficient was > 0.70. *FHR* Fetal Heart rate, *FSI* Fetal stress index, *SDNN* standard deviation of RR intervals, *RMSSD* root mean square of successive difference of RR intervals, *LF* low frequency, *HF* High frequency, *HFn.u.* normalized high frequency, *LTV* long term variability, *STV* short term variability, *AUC dec* deceleration area.

All analyses of the ECG and the RR series (space between two R waves of the QRS complex of the fetal ECG) were performed using the FSIrelecture© software package, which enables automatic data collection via cursor movements on the FHR (see Fig. 1—Data supplementary). These analyses were performed at the end of the baseline phase and at each of the stable periods (occurring every 20 min during the occlusion periods). The various parameter values recorded were:

- Time analysis: HRV time domain analyses included the root mean square value of successive differences (RMSSD) for the RR intervals and the standard deviation of the RR intervals (SDNN), computed over 540 RR intervals which correspond to approximately 3 min, considering a basal FHR of 180 bpm). RMSSD is related to parasympathetic nervous system activity, whereas SDNN evaluates ANS global activity^19^. Two additional specific fetal HRV indices were also tested: short-term variability (STV) and long-term variability (LTV). STV was computed over 1 min following a 4-Hz resampling of the RR series and was defined as the mean difference between successive 3.75-s periods for which the averaged RR interval epochs > 1 min (i.e., 16 epochs). LTV represented the difference between the maximum and minimum of the 16 epochs.
- Spectral analysis: Adult’s standards bandwidths are defined as 0.05–0.15 Hz for Low Frequency (LF) and 0.015–0.4 Hz for High Frequency (HF). However, there’s no consensus or standardization for specific fetal frequency bandwidths. In 2007, David et al. defined the LF bandwidth as LF 0.08–0.2 Hz and the HF bandwidth as 0.4–1.7 Hz^20^. In their review paper published in 2008, Van Laar et al., compared 6 studies on fetal HRV analysis with different bandwidths definitions^21^. 3/6 studies used 0.04–0.15 for the LF band definition. For these three studies, HF bandwidths were defined as 0.15–0.4 Hz, 0.15–1 Hz and > 0.15 Hz. In our study, we defined LF as 0.15–0.4 Hz and HF as > 0.15 Hz. Spectral HRV analyses were carried out after performing an 8-Hz RR series resampling using a Daubechies-4 wavelet transform^22^. We then computed the low-frequency (LF) component, 0.04–0.15 Hz, which is related to both sympathetic and parasympathetic activities, and is also associated with baroreflex activity. We also computed the high-frequency (HF) component, > 0.15 Hz (i.e. 0.15–4 Hz with an 8 Hz sampling rate), which is related only to the parasympathetic nervous system. HFnu was defined as HFnu = HF/(LF + HF).

FSI: Fetal Stress Index (FSI) reflects relative parasympathetic activity and has been previously described^16,23^. Briefly, the RR series was normalized and filtered to retain only the HF oscillation. The magnitude of these oscillations was then represented as the area between the local maximum envelope and the local minimum envelope. That is, the FSI measured the magnitude of the HF oscillation of a normalized RR series. The FSI therefore represents the numerical proportion of HF oscillations (between 0.15 and 4 Hz) within the variability as a whole, on a scale of 0–100. The average FSI was calculated post hoc as the average of the 4-min instantaneous FSI values at the end of the periods corresponding to a stable period. STV, LTV, HF, LF, HFn.u. and FSI were computed every second and averaged over 3 min. Differences in RMSSD, SDNN, STV, LTV, HF, HFnu, LF or FSI was defined as the percentage difference (increase or decrease) from the baseline phase and denoted ΔRMSSD, ΔSDNN, ΔSTV, ΔLTV, HF, ΔHFnu, ΔLF or ΔFSI.

#### Morphological analysis

Morphological analysis of decelerations included duration, amplitude (start to nadir), and slope velocity of the deceleration (amplitude/duration). Following Cahill et al., we studied the total deceleration area (AUC dec) defined by the sum of the areas of each deceleration, with each area estimated as ½ × duration × amplitude^24^. This analysis focused on the last deceleration before the stable period.

In total, 23 variables were studied. A descriptive analysis of these variables is given in Table 1.

**Table 1:** Characteristics of FHR recordings by acidosis or non-acidosis group.

Type of variables	pH groups	
	Non acidosis	Acidosis
	(n = 101)	(n = 29)
pH	7.28 [7.17 to 7.30]	7.05 [6.98 to 7.08]
**Fetal heart rate**		
FHRmean (bpm)	181 [169 to 187]	182 [164 to 195]
FHRmax	179 [169 to 190]	181 [164 to 196]
FHRmin	80 [73 to 96]	87 [68 to 96]
**Heart rate variability**		
SDNN	49.85 [36.69 to 65.26]	44.89 [28.97 to 56.56]
Delta-SDNN	28.24 [11.10 to 48.34]	23.02 [6.71 to 37.55]
RMSSD	19.95 [13.34 to 42.13]	23.80 [18.12 to 35.18]
Delta-RMSSD	8.82 [− 0.28 to 29.00]	9.17 [1.20 to 26.50]
LF	0.11 [0.05 to 0.18]	0.17 [0.10 to 0.31]
Delta-LF	0.01 [− 0.03 to 0.05]	0.02 [0.00 to 0.11]
HF	0.05 [0.02 to 0.11]	0.10 [0.04 to 0.17]
Delta-HF	0.01 [0.03 to 0.05]	0.02 [0.00 to 0.11]
HFnu	0.31 ± 0.09	0.35 ± 0.08
Delta-HFnu	− 0.00 ± 0.10	0.03 ± 0.08
FSI	57.59 ± 10.08	69.75 ± 10.22
Delta-FSI	− 0.82 [− 7.78 to 6.74]	4.14 [0.93 ; 15.24]
STV (ms)	3.79 [2.96 to 5.06]	4.67 [3.62 to 5.58]
Delta-STV	0.40 [− 0.80 to 1.93]	0.65 [− 0.36 to 2.04]
LTV (ms)	38.33 [31.27 to 48.65]	42.38 [29.67 to 52.49]
Delta-LTV	5.05 [− 7.35 to 16.04]	5.24 [− 11.4 to 14.04]
**Morphology of decelerations**		
Duration (s)	39 [27 to 52]	47 [28 to 68]
Amplitude (bpm)	95 [81 to 109]	103 [84 to 118]
Slope (bpm/s)	2.38 [1.77 to 3.72]	2.08 [1.73 to 3.71]
AUC dec	3 812 [1 958 to 61 433]	3 030 [1 728 to 5 476]

Values are expressed as mean ± standard-deviation or median [interquartile range].

*FHR* Fetal Heart rate, *FSI* Fetal stress index, *SDNN* standard deviation of RR intervals, *RMSSD* root mean square of successive difference of RR intervals, *LF* low frequency, *HF* high frequency, *LTV* long term variability, *STV* short term variability, *AUC dec* deceleration area.

### Statistical analysis

Quantitative variables are expressed as mean (standard deviation) in the case of normal distribution or median (interquartile range, IQR) otherwise. Normality of distributions was assessed using histograms and the Shapiro–Wilk test.

A principal component analysis (PCA) followed by a varimax rotation was applied on all the available variables to identify highly correlated variables. Pearson’s correlation coefficients between quantitative variables were described. Based on PCA results, Pearson’s correlation coefficients and clinician opinion, a set of uncorrelated variables was identified.

We first assessed the associations between all uncorrelated candidates predictors and the occurrence of acidosis using univariable logistic regressions and secondly, we used multivariable method to develop a predictive model of acidosis. For each continuous predictor, the log-linearity assumption was assessed using the restricted cubic spline functions and the absence of collinearity between variables was checked by calculating the variance inflation factors. When the log-linearity was rejected, variables were log-transformed or split into classes (with thresholds determined according to the median value of the variable). To account for the number of candidate predictors and limit the risk of over-optimism, this model was built using a bootstrap resampling (n = 500) with, in each sample, automated stepwise backward selection procedure (with a removal criteria of p-value > 0.05)including all uncorrelated candidate predictors^25^. The variable was kept in the final model if it was selected in at least 70% of these 500 analyses. The final predictive model was obtained by performing a logistic regression including the variables retained. We computed odds ratios (OR) and their 95% confidence intervals (CI). The predictive ability of the model was assessed by the area under the ROC (receiver operating characteristic) curve with its 95% confidence interval.

We also performed a decision-tree analysis using CHAID algorithm (Sipina software). Decision tree is a non linear (stepwise forward) multivariable analysis to predict a categorical outcome (in this study the acidosis status) from a set of continuous predictors. At the first step, the continuous variables are transformed into binary variables with the threshold maximizing the chi-square statistic. Then the most predictive variable according to the chi-square statistic is selected among the whole set of predictors. The process is repeated in each sub-population until no significant variable at 5% level for the chi-square is found. In parallel, we also developed a decision tree using the CART algorithm (package Rpart, R Software).

The performances of the different models were assessed by calculating the sensitivities (Se), specificities (Sp), positive and negative predictive values (PPV and NPV).

The analyses were conducted with the complete case sample.

Statistical testing was conducted at the two-tailed α-level of 0.05. Data were analyzed using the SAS software version 9.4 (SAS Institute, Cary, NC), the R software (R Core Team, 2019, version 3.6.1) and the Sipina software.

## Results

### Characteristics of the FHR recordings

Among the 130 FHR recordings, 29 were classified in the "acidosis" group and 101 in the "no acidosis" group. Table 1 describes the characteristics of the FHR recordings.

### Identification of uncorrelated variables

PCA with varimax rotation was used to identify a subset of uncorrelated variables. Seven factors, whose eigenvalues were greater than one, explained 84% of the variance. These seven factors were then rotated (varimax rotation) and then the variables showing a clear separation (each variable is associated with a single factor) and having correlations greater than 0.6 with the factors were selected. The correlations between the variables and the factors, are given in Table 2. Moreover, Pearson's correlations between all variables are shown in Fig. 1.

**Table 2:** Rotated factor loadings.

	Factor 1	Factor 2	Factor 3	Factor 4	Factor 5	Factor 6	Factor 7
FHR mean (bpm)	0.07	− 0.11	− 0.12	0.09	**0.86**	− 0.02	− 0.10
**FHR max**	0.03	− 0.05	− 0.14	0.19	**0.91**	0.02	0.12
**FHR min**	− 0.12	− 0.01	− 0.24	− 0.03	0.42	− 0.01	− **0.81**
**SDNN**	0.15	0.18	**0.80**	− 0.20	− 0.35	0.05	0.12
Delta SDNN	0.14	0.22	**0.87**	− 0.07	− 0.21	0.08	0.13
RMSSD	0.26	0.11	**0.86**	− 0.10	− 0.06	− 0.04	0.05
Delta RMSSD	0.24	0.16	**0.91**	0.07	0.14	− 0.03	0.003
**LF**	**0.96**	0.12	0.15	− 0.06	− 0.02	− 0.02	0.07
Delta LF	**0.95**	0.14	0.19	− 0.003	0.04	− 0.02	0.06
HF	**0.92**	0.19	0.16	0.10	0.003	− 0.02	0.06
Delta HF	**0.87**	0.22	0.24	0.22	0.12	0.02	0.04
HFNu	− 0.06	− 0.06	0.05	0.59	0.13	− 0.45	0.01
**Delta HFNu**	0.01	− 0.13	0.11	**0.77**	0.24	0.03	− 0.004
**FSI**	0.14	0.20	− 0.18	**0.72**	0.02	− 0.18	0.003
**Delta FSI**	0.10	− 0.01	− 0.24	**0.75**	0.02	0.17	0.16
**STV (ms)**	0.17	**0.90**	0.06	− 0.05	− 0.15	0.06	0.07
Delta STV	0.12	**0.92**	0.15	0.13	0.02	− 0.10	− 0.02
LTV (ms)	0.21	**0.87**	0.16	− 0.17	− 0.18	0.06	0.02
Delta LTV	0.15	**0.86**	0.24	0.05	0.06	− 0.12	− 0.09
**Amplitude**	0.13	− 0.05	0.07	0.20	0.50	0.04	**0.80**
**Duration (sec)**	0.06	0.01	0.04	0.21	0.08	− **0.85**	0.01
**Slope (bpm/sec)**	0.03	0.01	0.01	− 0.09	0.22	**0.77**	0.43
**AUC dec**	− 0.02	− 0.11	0.12	0.36	− 0.04	**0.68**	− 0.23

Absolute values higher than 0.6 are in bold font. Variables retained for the construction of the multivariable models are indicated in bold font.

*FHR* Fetal Heart rate, *FSI* Fetal stress index, *SDNN* standard deviation of RR intervals, *RMSSD* root mean square of successive difference of RR intervals, *LF* low frequency, *HF* high frequency;

LF, Delta LF, HF and Delta HF are highly correlated with the first factor, STV, Delta STV, LTV and Delta LTV with the second factor, SDNN, Delta SDNN, RMSSD and Delta RMSSD with third factor, Delta HFn.u., FSI and Delta FSI with the fourth factor, FHR mean and FHR max with the fifth factor, Duration, Slope and AUC dec with the sixth factor, and FHR min and amplitude with the seventh factor. Among the variables highly associated with the seven factors, we noticed five sets of strongly correlated variables. Indeed, variables correlated with factor 4 (Delta HFnu FSI and Delta FSI) and with factor 7 (FC min and Amplitude) didn’t show any strong correlation between each other and were keep for the multivariate analysis. To avoid multicollinearity problems in further multivariable analysis, some variables from the five sets of strongly correlated variables were excluded based on clinical expertise. Among LF, delta LF, HF and delta HF, LF was retained because HF represents the parasympathetic system, which is already represented in terms of Delta HFn.u. and FSI. Among STV, delta STV, LTV and delta LTV, STV was retained because of its more frequent use in clinical practice. Among SDNN, delta SDNN, RMSSD and Delta RMSSD, SDNN was retained because it represents the whole variability whereas RMSSD represents the parasympathetic activity which is already estimated through FSI, DeltaFSI and DeltaHFn.u. Moreover, the baseline condition is not always known in clinical practice and therefore the value of the delta is not always available. For Factor 5, the variable FHR max is retained. [For factor 6, there were a strong correlation between Duration and Slope and no correlation between AUC dec and other parameters. We therefore retained Duration and AUC dec for this factor. A total of 11 variables were then retained (these variables are indicated in bold font in Table 2).

### Construction of acidosis prediction models

**Figure 2:**
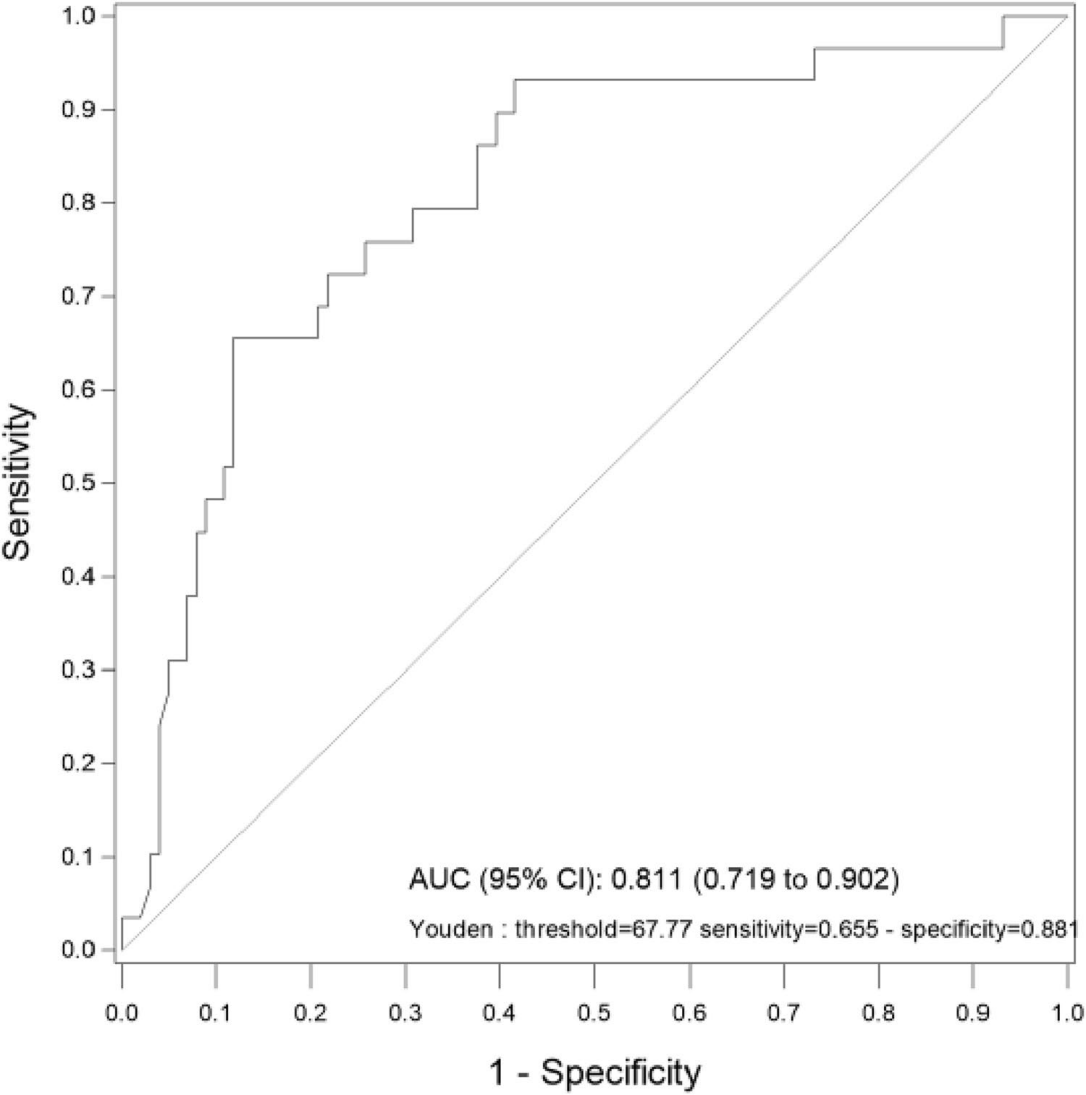
Ability of FSI to predict acidosis. *AUC* area under the curve, *CI* confidence interval.

Univariate logistic regressions are presented in Table 3. FSI was the only significant variable for predicting acidosis with p < 0.001, OR (CI 95%) 1.12 (1.07–1.17). Using multivariate logistic regression model with bootstrap resampling and stepwise backward selection, performed on the 11 variables, only FSI was selected. The AUC of this variable was 0.81, which is considered as a good discriminant power. Considering a threshold of 67.8, we obtained a sensitivity of 0.66, specificity of 0.88, PPV of 0.61, and NPV of 0.90 (see Fig. 2).

**Table 3:**
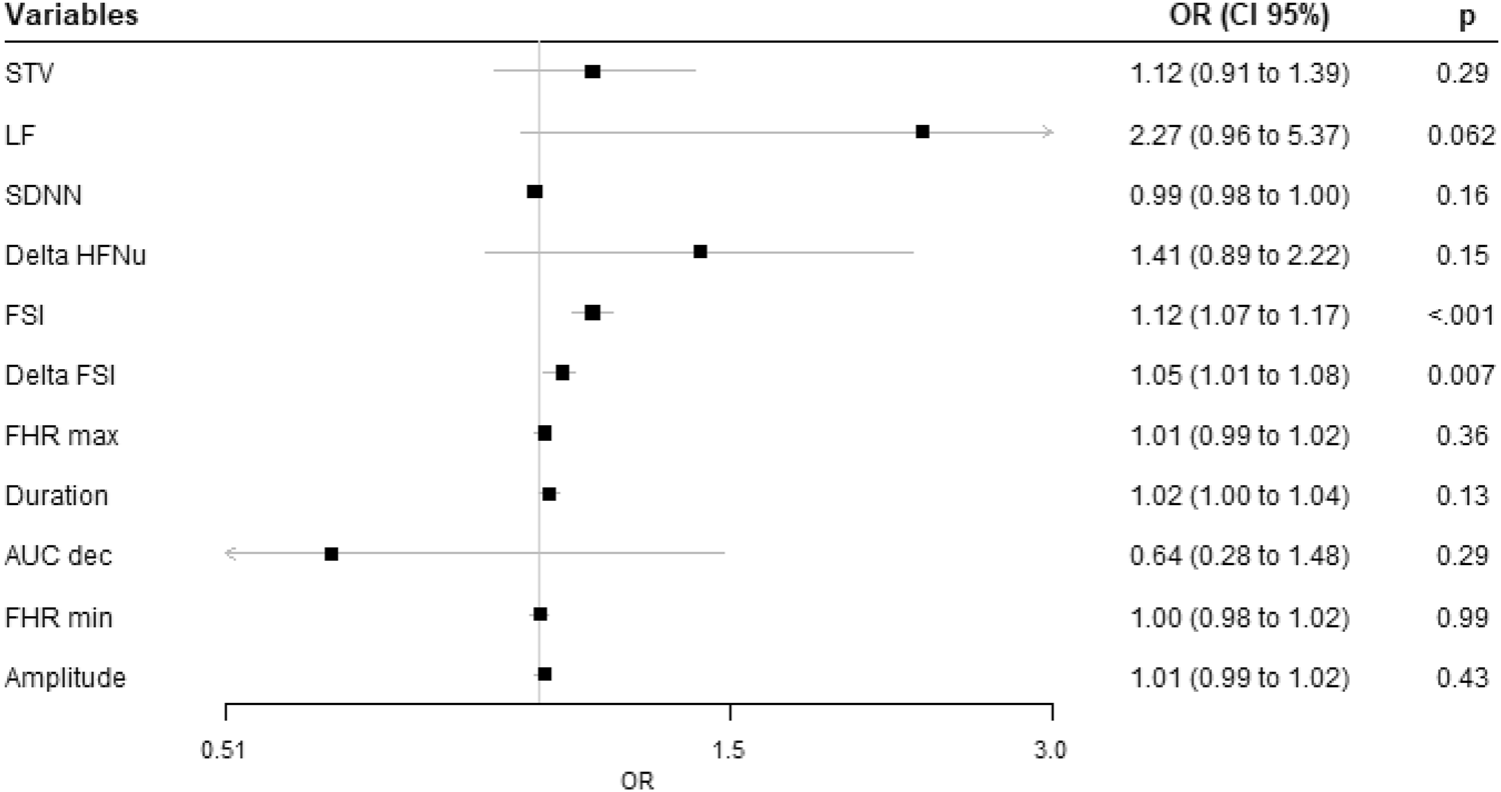
Results of the univariate analysis for the prediction of acidosis.

Statistical significance was accepted at p < 0.05.

*FHR* Fetal Heart rate, *FSI* Fetal stress index, *SDNN* standard deviation of RR intervals, *RMSSD* root mean square of successive difference of RR intervals, *LF* low frequency, *HF* high frequency, *LTV* long term variability, *STV* short term variability, *AUC* deceleration area, *OR* odds ratio, with odds-ratios calculated per 0.1 increase for Delta HFnu, *CI* confidence interval.

**Figure 3:**
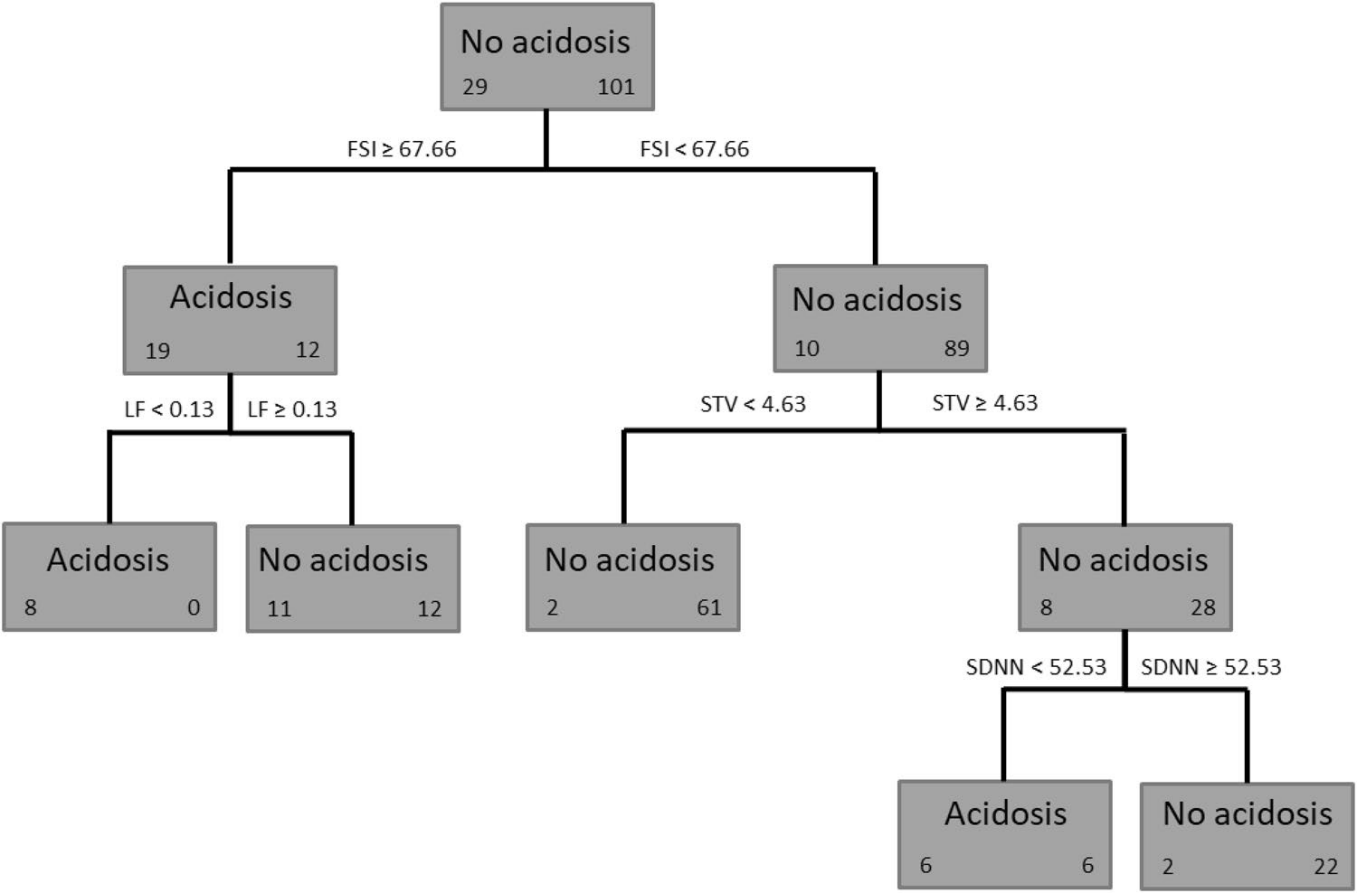
Decision tree obtained using CHAID algorithm. For each leaf, we indicate in bold font the predicted class, on the left, the number of individuals in the acidosis class and, on the right, the number of individuals in the no acidosis class. *FSI* Fetal stress index, *LF* low frequency, *STV* short term variations, *SDNN* standard deviation of RR intervals. This model correctly classified 95/101 recordings as non-acidosis and 14/29 recordings as acidosis, giving a sensitivity of 0.48, specificity of 0.94, PPV of 0.70, and NPV of 0.86.

**Figure 4:**
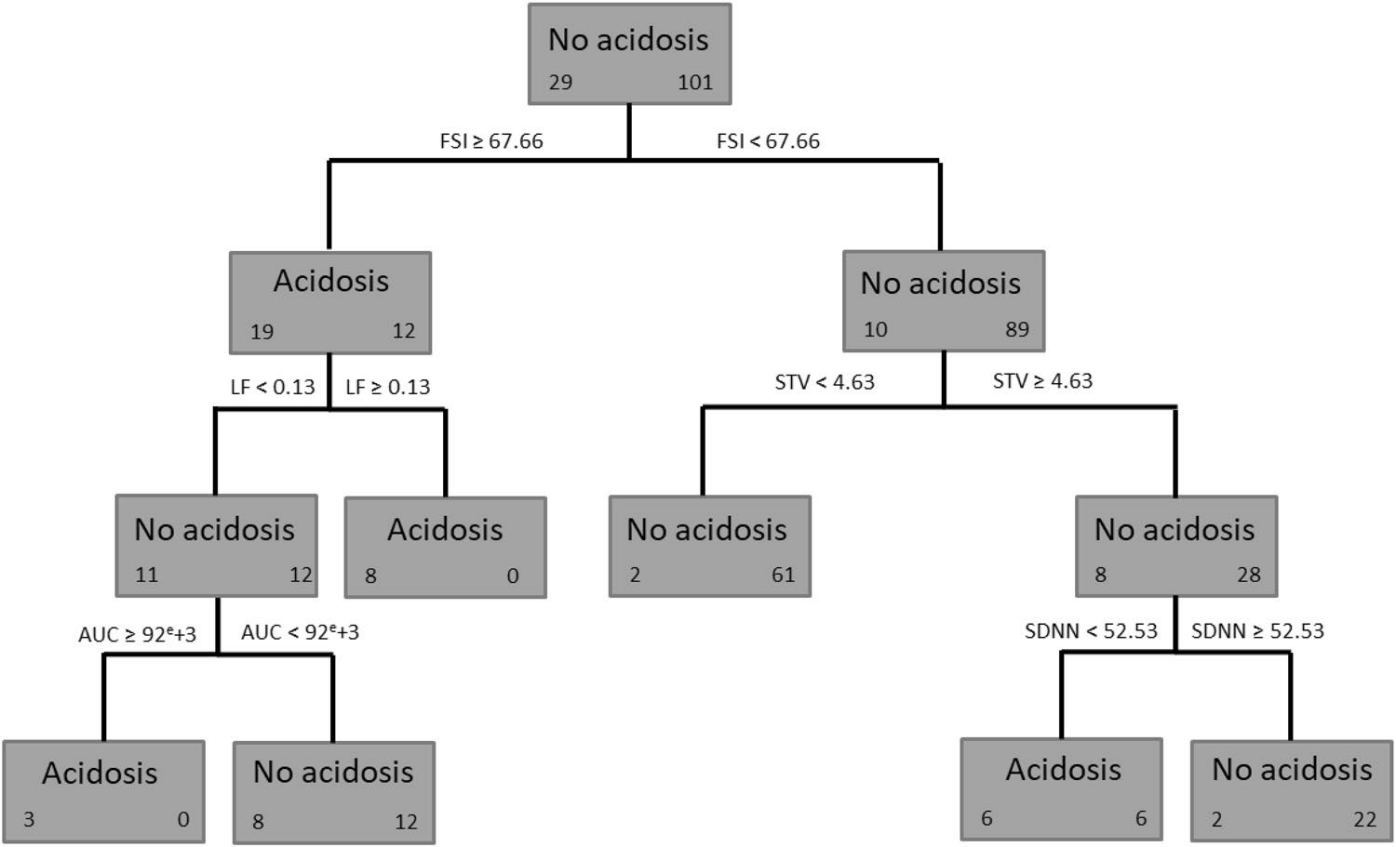
Decision tree obtained using CART algorithm. For each leaf, we indicate in bold font the predicted class, on the left, the number of individuals in the acidosis class and, on the right, the number of individuals in the no acidosis class. *FSI* Fetal stress index, *LF* low frequency, *STV* short term variations, *SDNN* standard deviation of RR intervals, *AUC* deceleration area. This model correctly classified 95/101 non-acidosis and 17/29 acidosis, i.e., a sensitivity of 0.59, a specificity of 0.94, a PPV of 0.74 and an NPV of 0.89.

Two algorithms of decision trees were applied, namely CHAID and CART algorithms (see Figs. 3, 4). For each tree, the 11 uncorrelated variables were used.

Using CHAID algorithm, the first variable that classified recordings as "acidosis" or "no acidosis" was FSI, followed by STV, LF and SDNN (see Fig. 3). This model correctly classified 95/101 recordings as "no acidosis" and 14/29 recordings as "acidosis", giving a sensitivity of 0.48, a specificity of 0.94, a PPV of 0.70, and an NPV of 0.86.

Using CART algorithm, the first variable to classify the recordings as "acidosis" or "no acidosis" was FSI, followed by STV and LF, and finally AUC and SDNN (see Fig. 4). This model correctly classified 95/101 as "no acidosis" and 17/29 as "acidosis", i.e., a sensitivity of 0.59, a specificity of 0.94, a PPV of 0.74 and an NPV of 0.89.

## Discussion

### Main results

Our models involving HRV variables in an experimental model of ewe fetus do not appear to predict fetal acidosis optimally. However, despite the low sensitivity, all the models showed very good specificity.

The CART decision tree approach seemed to be more predictive of acidosis and was closer to clinical-decision reasoning. We noted that the most discriminating of the selected variables were those from HRV analysis, rather than the FHR variables usually preferred in classical clinical practice (deceleration morphology and heart rate). Finally, FSI emerged as the most discriminating variable, underlining the contribution of the parasympathetic system in the regulation of fetal adaptation mechanisms with respect to hypoxia.

### Interpretation

In our studies, we demonstrated that fetal acidosis was associated with an increase fetal HRV. Indeed, Table 1 shows higher values in most of the HRV parameters in the “acidosis” group when compared to “no-acidosis” group. Even STV and LTV which are used in clinical practice were higher for pH values lower than 7.10. STV is used in clinical practice for antepartum fetal monitoring and a reduce STV is interpreted as a risk of fetal compromise. However, several studies demonstrated that, during labor neonatal acidosis is associated with higher STV values^26,27^.

Despite the increasing application of AI to medicine in recent years, the literature in the area of FHR interpretation remains scarce. Several authors have studied the prediction of fetal acidosis using automated or ML systems. Most of the studies comparing visual analysis of the intrapartum FHR with interpretation by an automated system did not show any significant superiority of the automated analysis with respect to neonatal prognosis. Only the retrospective before/after study of Lopes-Pereira et al. using SisPorto showed a reduction in the risk of HIE^12^. In their study of 38,446 deliveries, they found a significant decrease in the number of cases per 1000 births of HIE since the introduction of the SisPorto system over the period 2001–2003, with 5.3% [95% CI (4.0–7.0)], against the period 2004–2014, with 2.2% [95% CI (1.7–2.8)], and RR = 0.42 [95% CI (0.29–0.61)]. They also found a slight reduction in emergency cesarean deliveries, with 21.6% [95% CI (20.7–22.4)] against 19.2% [95% CI (18.8–19.7)] and RR = 0.91 [95% CI (0.87–0.95)]. Finally, they found an increase in the rate of instrumental deliveries, with 19.5% [95% CI (18.7–20.3)] against 21.4% [95% CI (21.0–21.9)], RR = 1.07 [95% CI (1.02–1.13)]. Furthermore, these existing systems, developed using SisPorto and INFANT, are not compatible with some existing CTGs, which may require an extra cost, and which would limit their immediate adoption^28^.

In this study, we evaluated the ability of ML system to predict fetal acidosis. No complex ML system could be used in our model because of the low number of "acidosis" events (we had only 29 such events). Several authors studied ML systems for acidosis prediction. Typically, they started with the CTG FHR recordings, analyzed them via various ML and deep-learning systems, and compared them against visual analysis with respect to the prediction of pathological FHR or pH at birth^29–35^. For example, Zhao et al. studied several FHR features based on the 2015 FIGO recommendations and integrated them into various ML algorithms to predict acidosis at birth^3^. The algorithms included decision trees, support vector machines (SVMs), and AdaBoost^31^. They analyzed 552 intrapartum FHR recordings obtained via CTG from 2009 to 2012, which were classified into two groups according to neonatal arterial pH (Czech Republic database, Brno University Hospital)^36^. In the pH < 7.15 group, 105 recordings were analyzed compared with 447 in the pH > 7.15 group. The variables included in their ML model were analyzed according to FIGO classification (baseline, decelerations), temporal analysis (RR, NN, SDNN, RMSSD, STV), frequency analysis (HF, LF, and VLF), and nonlinear analysis (ApEn, SampEn, LZC, AAC, ADC, APRS, DPRS, SD1, SD2). AdaBoost performed best, offering good predictions of acidosis with Se of 92% and a Sp of 90%. The same team also considered neural networks, using the same recordings, finding better predictions of acidosis (pH < 7.15) than the more conventional ML systems, with an accuracy of 98.69%, Se of 99.28%, and Sp of 98.10%^37^. In 2019, Hoodbhoy et al. compared 10 different ML algorithms for the interpretation of 2126 CTG recordings from patients in the third trimester of pregnancy^35^. They compared the performance of ML against visual analysis of the FHR performed by three expert obstetricians in classifying the FHR as normal, suspicious, or pathological. Of the 2126 recordings, 70% were classified as normal, 20% as suspicious, and 10% as pathological. Of the 10 ML models studied, XGBoost, decision trees, and Random Forest achieved high accuracy (> 96%) and Se (> 99%) with respect to predicting suspicious and pathological FHRs. When considering only suspect FHRs, XGBoost had a lower Se (73%), but overall, on all records, XGBoost was the most accurate (93%). Therefore, ML systems seem to perform well with respect to the prediction of neonatal acidosis. None of these studies evaluated the ability to predict intrapartum acidosis. Our model was less accurate than those using complex ML algorithms. However, our limited sample size didn’t allow the use of AdaBoost, XGBoost or neural network.

In our experimental study, we noted the superiority of FSI over other possible markers. In reflecting the fluctuations of the parasympathetic system, our results corroborate the importance of the autonomic nervous system shown in previous work^14,38–40^. In addition, a more physiological approach to the interpretation of the FHR during labor has been proposed by many authors^41–44^.

Most of this knowledge comes from experimental studies, particularly the studies carried out by the team of Lear et al.^39,45–47^. In their various experimental studies, they emphasized the importance of the parasympathetic system in fetal adaptation to hypoxia via activation of the chemoreflex^46^. When blocking the parasympathetic system, by injection of atropine or by cervical vagotomy, in an experimental model of repeated UCO in the ewe fetus, they showed an overall decrease in HRV and a significant increase in FHR and MAP just after occlusion in the atropine and vagotomy group compared with a control group. Again, this showed the importance of the parasympathetic system in regulating the fetal adaptation mechanisms with respect to hypoxia^39^.

### Strengths, limitations and perspectives

In our study, HRV analysis was not continuously evaluated but on 5 min periods every 20 min. In addition, the morphological analysis, and area of decelerations was only performed on single decelerations before those periods. This method may have missed subtle changes that may have been seen had more periods/decelerations been sampled.

Most studies found in the literature on automated system use per partum CTG recordings, which should not be expected to be sufficiently precise to enable accurate analysis of most of the HRV indexes, particularly those exploring the HF content (i.e., the parasympathetic nervous system)^21^. Our study has the advantage of using ECG recordings that allow the inclusion of an HRV analysis.

Though our model reproduced the cord compression which occur during labor, the experimentation wasn’t preformed in real labor conditions. Moreover, we investigated fetuses at 124 days of gestation (i.e. 0.85 of the term to avoid any risk of birth between surgical preparation and experimentation) whereas most of the studies in human fetuses were performed in the term period. On the other hand, such an experimental model allows a repeated evaluation of the fetal acid–base status by taking regular samples during the cord occlusion protocol, thereby enabling identification of the precise moment that acidosis begins.

Though it presented a low sensitivity, our experimental model demonstrated that HRV indexes multivariate analysis showed good specificity, PPV and NPV to distinguished pH ≤ 7.10 and pH > 7.10. This experimental study confirmed the results from a study performed on human fetuses’ CTG records where Gatellier et al. demonstrated that a multivariate model including FSI, STV and LTV allowed better discrimination than the FIGO classification with ROC AUC = 0.713 and 0.569 respectively^43^. However, even if it performed better than FIGO, this model presented a low sensitivity/specificity.

Any interpretation of our results must consider the low number of events in the "acidosis" group. It would be interesting to test the various models on a larger dataset and to use more complex ML models. However, animal experimentation, limited in the number of animals because of ethical considerations, does not lend itself well to the realization of large-scale tests. The use of such systems in humans will therefore be interesting, but will require high-quality fetal ECGs to be obtained. Indeed, the commonly used Doppler ultrasound technique used for human fetuses monitoring does not reflect the real beat-to-beat variability, which must be known for efficient HRV analysis. In clinical practice, HRV estimation will require the use of scalp electrodes or transabdominal fetal ECG which now allow an accurate beat-to-beat noninvasive FHR computation^48^.

However, in a previous analysis performed on human fetuses CTG records, we demonstrated that a multivariate model including FSI, STV and LTV allowed better discrimination than the FIGO classification^43^.

The application of our model to human fetuses should also require to take into account several influencing factors which may influence the autonomic nervous system like the gestational age, the fetal state of activity (sleep state) or the course periodic uterine contractions during labor. All these potential confounding factors should be included in the multiparametric model.

Here, we have used fetal pH as the gold standard for classifying our recordings. It is increasingly noted in the literature that fetal pH is not a good predictive marker of AIE^49–51^. The HRV multivariate analysis for the prediction of HIE lesions rather than acidosis will therefore be evaluated in future work. The inclusion of HRV analysis in predicting HIE lesions will have its place in this model. It has been shown in stroke patients that changes in HRV, reflecting ANS dysfunction, are associated with an increased risk of mortality and neurological morbidity^52,53^, although changes in HRV during the early phases of stroke have yet to be studied. However, Block et al. are conducting a prospective study on the use of AI for the early detection of ischemic brain injury in adult patients who have undergone carotid endarterectomy or cerebral embolectomy^54^. They intend to use EEG and HRV markers in their AI model.

## Conclusion

The use of automated system for FHR interpretation has the main advantage of reducing interobserver variability and could improve fetal well-being and neonatal prognosis. It could also have important medico–legal implications by providing an objective and unbiased analysis of the FHR. However, this study just constitutes a preliminary experimental study and results need to be confirmed in a real clinical setting in human fetuses.

## Supplementary Information

Supplementary Information.
