# Supplementary material for: Heart rate markers for prediction of fetal acidosis in an experimental study on fetal sheep
Source: Sci Rep. 2022 Jun 23;12:10615. doi: 10.1038/s41598-022-14727-4 (PMC9226053; doi:10.1038/s41598-022-14727-4)
Supplement: Supplementary file 1 — Supplementary Information. [file 41598_2022_14727_MOESM1_ESM.docx]

Data Supplementary:

Figure 1 - FSIrelecture © software.

*
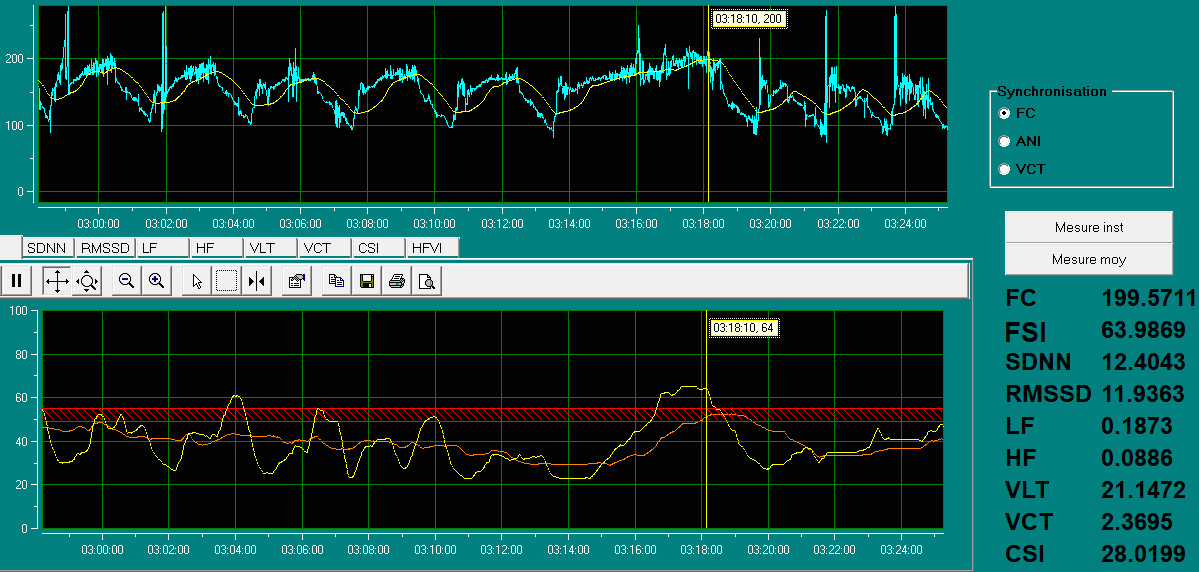
*

*FC=HR=Heart rate; FSI=Fetal stress index; SDNN= standard deviation of RR intervals; RMSSD= root mean square of successive difference of RR intervals; LF=Low frequency; HF=High frequency; VLT=LTV=long term variability; VCT=STV=short term variability*

*The FSI, SDNN, RMSSD, LF, HF, LTV, STV are measured at the end of a plateau between cord occlusions resulting in slowing of the fetal heart rate (^2nd^ window).*
